# Dissemination of multidrug-resistant tuberculosis in a patient with acute HIV infection

**DOI:** 10.1186/1471-2334-14-462

**Published:** 2014-08-26

**Authors:** Kyung Mo Yoo, Eun-Jeong Joo, Joon-Sup Yeom, Seoung-Wan Chae, Shin Yeoung Lee, Ki Joong Han

**Affiliations:** Department of Internal Medicine, Kangbuk Samsung Hospital, Sungkyunkwan University School of Medicine, Seoul, South Korea; Department of Pathology, Kangbuk Samsung Hospital, Sungkyunkwan University School of Medicine, Seoul, South Korea

**Keywords:** HIV, Multidrug-resistant, Tuberculosis

## Abstract

**Background:**

Opportunistic infections are relatively rare in early human immunodeficiency virus infection, especially infection with *Mycobacterium tuberculosis*. Here, we report a patient who presented with acute human immunodeficiency virus and multidrug-resistant *M. tuberculosis* co-infections.

**Case presentation:**

A 27-year-old homosexual male was admitted for fever, cough, and hepatitis. At the time of admission, the p24 antigen was detected in his serum, indicating that he had an acute human immunodeficiency virus infection. He was also diagnosed with disseminated tuberculosis spreading to the lung and skin. Anti-tuberculosis medication had been started earlier with one-week intervals of highly active antiretroviral therapy. Despite prolonged anti-tuberculosis treatment, the patient developed tuberculous meningitis on the 50^th^ day of admission. Multidrug-resistant tuberculosis was cultured from his sputum and cerebrospinal fluid. The patient was successfully treated with second line anti-tuberculosis medication and antiretroviral treatment.

**Conclusion:**

This is the first case of acute human immunodeficiency virus and multi drug-resistance tuberculosis co-infections. This case indicates that tuberculosis infection should be considered even in patients with acute human immunodeficiency virus infection.

**Electronic supplementary material:**

The online version of this article (doi:10.1186/1471-2334-14-462) contains supplementary material, which is available to authorized users.

## Background

Although *M. tuberculosis* (TB) is the major opportunistic infection in human immunodeficiency virus (HIV)-infected patients, conjunction with acute HIV infection is very rare. The only one case of TB infection during the acute HIV infection was reported [1]. multi drug-resistance (MDR)-TB in acute HIV infection has not been reported previously to our knowledge.

We describe a case of subsequent dissemination of MDR-TB to the lung, skin and central nervous system (CNS) in a patient with acute HIV infection. The patient was successfully treated with second-line anti-TB medication with the use of antiretroviral therapy (ART).

## Case presentation

A 27-year-old homosexual male was admitted for fever, cough and nausea lasting for 2 weeks. He had no history of underlying disease. He had unprotected homosexual contact with multiple partners several weeks prior to presentation. Initial physical examination revealed a temperature of 37.5°C, otherwise stable. His total white blood cell count was 1800 cells/mm^3^, with hemoglobin level and platelet count of 15.7 g/dl and 380,000/mm^3^. Serum aspartate transaminase and alanine aminotransferase was 199 IU/mL and 191 IU/mL. Chest x-ray showed no active lung lesions.

**Figure 1:**
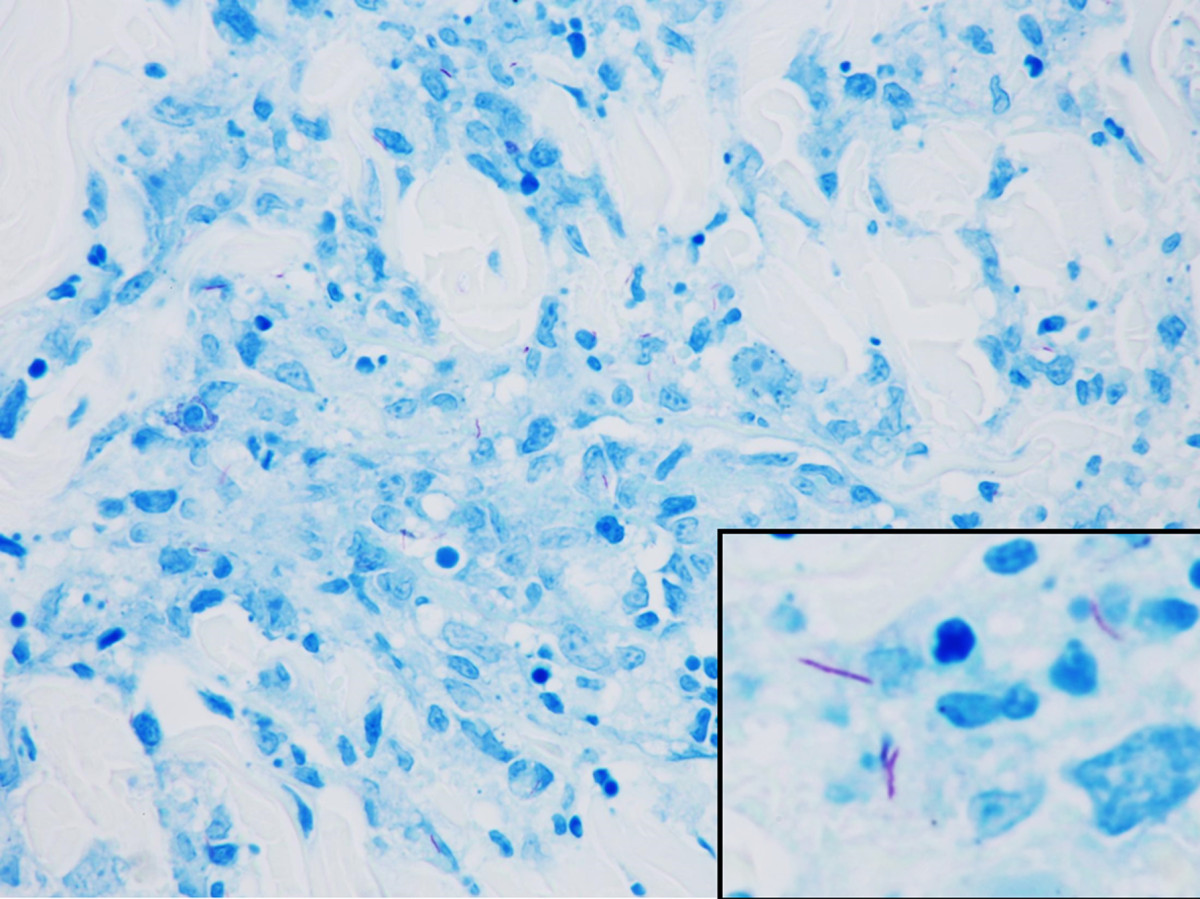
**Acid fast stain on section of skin lesion reveals scattered positive bacilli (inlet) (AFB stain x400).**

On the fifth day of admission, the patient developed fever with a body temperature of 40.0°C, whole body skin rash and cough. A fourth-generation screening HIV test for the detection of p24 antigen and HIV 1/2 antibodies (Abbott Molecular inc., Chicago, Illinois) was positive, although it had been negative when he was checked 6 months prior to presentation. Chest Computed Tomography scan showed hematogenous spreading pulmonary nodules. We performed a skin punch biopsy of the rash on the patient’s left shoulder. AFB staining of the skin specimen showed multiple positive bacilli, which was suggestive of mycobacterial infection on the skin (Figure 1). Additional tests of the skin tissue were positive for *M. tuberculosis* by polymerase chain reaction (PCR). Furthermore, TB-PCR was also positive in sputum. On hospital day 14, we started the patient on anti-TB medication including isoniazide, rifampicin, ethambutol, and pyrazinamide. During that period of time, Western Blot results for HIV were positive in the gp160 and p24 bands. Measurements of HIV-1 RNA plasma levels by quantitative real-time PCR (COBAS TaqMan® HIV-1 Test, Roche Diagnostics, Mannheim, Germany) were greater than 10,000,000 copies/ml. CD4 T-cell counts at the same time were 55 cells/μL. The CD4 cells accounted for 33.5% of lymphocytes. The ratio of CD4/CD8 was 0.55.

Despite anti-TB treatment, the patient’s condition deteriorated, with ongoing fever and confused mental state. On hospital day 18, after four days of anti-TB treatment, he presented with lethargic state and cardiopulmonary instability with systolic blood pressure of 78 mmHg, and oxygen saturation level of 87% on reservoir mask. He was transferred to the intensive care unit (ICU). Follow-up chest CT showed aggravated hematogenous spreading nodules. The cerebrospinal fluid (CSF) was sterile. The growth of bacteria including *M. tuberculosis* was not observed in blood, sputum and urine cultures. Serologic tests for toxoplasma, cytomegalovirus, herpes simplex virus and Epstein-Barr virus were all negative.

On the 21^st^ day after admission, we started ART with efavirenz, lamivudine, and abacavir within 2 weeks of anti-TB therapy, because he had CD4 T-cell counts around 50 cells/μL and severe organ dysfunction (http://aidsinfo.nih.gov/guidelines).

The HIV-1 RNA viral load at this time was decreased to 724 copies/mL, but CD4+ T cell counts remained low, 45 cells/μL. Growth of *M. tuberculosis* in sputum was observed. Rapid molecular testing to detect drug-resistant tuberculosis (Genotype® MTBDRplus, Hain Lifescience GmbH, Nehren, Germany) revealed isoniazid-resistance and rifampin-susceptibility. Levofloxacin and amikacin were added to the first line anti-TB medication. After 20 days of ICU care, the patient was transferred to the general ward.

On the 50^th^ day of admission, the patient’s body temperature increased to 39.7°C. Lumbar puncture was performed due to newly developed severe headache and vomiting. CSF analysis showed that leukocytes were 1,258/mm3 with 94% poly-dominant cells. An *M. tuberculosis* PCR test of the CSF fluid was positive. Due to concerns regarding treatment failure of the anti-TB medication and the development of immune reconstitution inflammatory syndrome (IRIS), the anti-TB regimen was modified to include cycloserin, prothionamide, levofloxacin, rifabutin and amikacin and dexamethasone was added for the management of IRIS.

After 80 days of admission, drug susceptibility testing of sputum TB indicated MDR-TB that showed resistance to isoniazid, rifampicin and quinolone and sensitivity to rifabutin, ethambutol, pyrazinamide, amikacin, cycloserin, prothionamide and kanamycin. Drug susceptibility test (DST) was performed using the conventional absolute concentration method with Löwenstein-Jensen medium at the Korean Institute of Tuberculosis, the supranational reference laboratory for mycobacterial culture and DSTs [2]. Antimicrobial susceptibility tests were performed by the recommendations of the Clinical and Laboratory Standards Institute [3]. The results of susceptibility tests in sputum led us to re-modify the patient’s anti-TB medication regimen to rifabutin, ethambutol, pyrazinamide, PAS, cycloserin, prothionamide and kanamycin. After 30 days of anti-TB treatment with re-modified regimens, the additional resistance to ethambutol and pyrazinamide for TB was reported in the result of tuberuclosis DST in CSF, compared to that in sputum. We subsequently added amoxicillin-clavulanate and excluded ethambutol and pyrazinamide after DST result of CSF was identified.

Finally, 120 days after admission, 40 days since the re-modified TB medication was started according to DST results in sputum, the patient had recovered and exhibited no fever. Chest X-ray revealed near complete regression of infiltration, and follow-up sputum AFB culture was negative. CSF analysis showed sterile findings. The patient was subsequently discharged from the hospital. Five months after having started ART medication, his HIV RNA load was declined to 22 copies/mL and his CD4 count had increased to 510 cells/μL. At the time of writing, the patient was maintaining his anti-TB medications with prothionamide, cycloserine, amoxicillin-clavulanate, PAS and kanamycin and ART medication with lamivudine, abacavir, and efavirenz.

## Conclusions

Acute/early HIV infection is referred to as primary HIV infection or seroconversion illness. Patients observed at the time of seroconversion have been found to present with symptoms ranging from flu-like illness to encephalopathy, 2–3 weeks after acquisition of virus. Most symptoms usually resolve spontaneously. In the seroconversion state, there is sometimes a high rate of viral replication, leading to a rise in HIV viral load, and concomitant immune-suppression due to rapid loss of CD4+ T cells [4]. Opportunistic infections rarely occur in acute HIV infected patients who are in a transient CD4+ lymphocytopenia state. Esophageal candidiasis, toxoplasmic encephalitis, *Pneumocystis jirovecii* pneumonia, and cytomegaloviral disease have previously been reported [5–11].

In contrast to other HIV-associated opportunistic infections, TB can occur in HIV-infected patients at any level of immunodeficiency, regardless of CD4+ T cell counts. However, TB rarely occurs in acute HIV infection with only one case active pulmonary TB during acute HIV infection previously reported in the literature [1]. In our case, the patient suffered from flu-like symptoms and also exhibited the clinical findings of acute HIV infection. His HIV-1 RNA viral load, which is the most sensitive marker for acute HIV infection, was markedly elevated, while an HIV antigen/antibody test had been negative which had been tested 6 months prior. His Western Blot results for the HIV test were only positive in the gp160 and p24 bands, indicating that our patient suffered from acute HIV infection rather than latent AIDS.

In this case, DST for TB in sputum revealed the patient was infected with an MDR pathogen 80 days after admission. DST was performed by conventional absolute concentration method in Löwenstein-Jensen media at the Korean Institute of Tuberculosis. The conventional DST method usually took longer than two months to execute [12], so it could prolong the diagnosis and treatment of MDR pathogen. Recently, rapid direct susceptibility tests such as Genotype MTBDRplus assay were found to be useful to detect MDR-TB earlier [13]. Interestingly, additional resistance to ethambutol and pyrazinamide of TB was detected in DST result from CSF, which was acquired after 30 days of anti-TB medication. The acquisition of additional resistance during treatment might be associated with baseline resistance to 1^st^-line drugs, higher degree of lung pathology, and HIV co-infection [14]. In addition, it might be due to genomic evolution of serial clinical strains in patient under therapy. Recent studies applying genome sequencing tests revealed the mixture of different subpopulations and the stepwise acquisition of resistance conferring mutations selecting for the most resistant phenotype in the same patients [15, 16].

In this case, drug susceptibility tests of TB in sputum and CSF revealed the patient was infected with an MDR pathogen. To the best of our knowledge, this is the first case of acute HIV and MDR-TB co-infections. MDR-TB is caused by strains of *M. tuberculosis* that are resistant to isoniazid and rifampicin, with or without resistance to other anti tuberculous agents. As a result, this form of the disease is more difficult to treat than ordinary TB and requires up to 2 years of multidrug treatment. The relationship between MDR-TB and HIV is not yet clearly understood, but many studies have found strongly increased risks for MDR-TB among patients co-infected with HIV [17, 18]. A recent systemic review and meta-analysis showed that HIV significantly increases the risk of MDR TB. HIV/MDR TB co-infection has been associated with high mortality rates due to greater degrees of immunosuppression and treatment failure [19]. CNS/meningeal involvement of TB in HIV-infected patients is usually observed in AIDS-defining condition [20]. Disseminated military MDR-TB with cutaneous and meningeal expressions, during acute stage of HIV infection in this case, might be attributed to his low CD4+ cell counts.

Both TB and HIV have profound effects on the immune system, but the mechanisms behind the breakdown of the immune defense of associated with co-infection are not fully understood. HIV infection interferes with generation of effector memory CD4+ T cells that migrate to the lung (the primary site of M. TB infection), and M. TB specific CD4+ T-cell responses are selectively depleted, further reducing the immune system’s ability to control TB [17, 21]. Other reports also showed that most opportunistic infections co-existing acute HIV infection occurred when CD4+ T cell counts temporarily dropped [6, 8, 22]. Moreover, the granuloma formation, which prevents the spread of TB, may fail in HIV-infected patients, probably due to the deaths of CD4+ T cells in the granuloma resulting in disruption of granuloma structure [17, 20]. In our case, the patient’s initial CD4+ T cell counts were 55 cells/μL; this depletion of M. TB specific CD4+ T cells may contribute to reduced immune-response for tuberculosis in the lung. Furthermore, lack of the granuloma formation due to reduced T cell-response may result in subsequent dissemination form the lung to the skin and CNS. In this case, however, it is difficult to distinguish whether dissemination of TB occurred as a result of primary infection or reactivation of latent infection during the acute HIV infection stage. The reactivation of latent TB could be suggested as another possible contributor to the development of TB [23]. Especially during acute HIV infection, regulatory T cells (Tregs) are upregulated, but HIV can target CD4+ Treg cells for infection, and Treg cells can suppress ant-HIV immunity, which may together promote an increase in acute viremia and infected cells [24]. High levels of Treg as well as numerical deficit of CD4+ T cells during acute phase of HIV infection imply immune dysregulation and this may have favoured the reactivation of latent TB [1].

In conclusions, we described a patient with both acute HIV infection and MDR-TB disseminated infection. The patient suffered from a life-threatening course while in the hospital, but was ultimately treated successfully with ART and second line anti-TB medications. This case emphasizes the fact that physicians must be aware that serious opportunistic infections including MDR-TB can occur during the acute phase of HIV infection.

### Consent

Written informed consent was obtained from the patient for publication of this Case report. A copy of the written consent is available for review by the Editor of this journal.

## Authors’ original submitted files for images

Below are the links to the authors’ original submitted files for images. Authors’ original file for figure 1

## Abbreviations

MDR: Multi drug-resistance
HIV: acute human immunodeficiency virus
M. Tb: *Mycobacterium tuberculosis*
CNS: Central nervous system
ART: Antiretroviral therapy
PCR: Polymerase chain reaction
CSF: Cerebrospinal fluid
IRIS: Immune reconstitution inflammatory syndrome
DST: Drug susceptibility test.

## References

[1] Sued O, Quiroga MF, Socias ME, Turk G, Salomon H, Cahn P (2011). Acute HIV seroconversion presenting with active tuberculosis and associated with high levels of T-regulatory cells. Viral Immunol.

[2] Kim SJ (2005). Drug-susceptibility testing in tuberculosis: methods and reliability of results. Eur Respir J.

[3] Wayne P (2011). Clinical and Laboratory Standards Institute (CLSI): Susceptibility Testing of Mycobacteria, Nocardiae, and Other Aerobic Actinomycetes; Approved Standard— Second. M24-A2 edition. Susceptibility Testing of Mycobacterium, Nocardiae, and other Aerobic Actinomycetes; Approved Standard-Second Edition.

[4] Streeck H, Nixon DF (2010). T cell immunity in acute HIV-1 infection. J Infect Dis.

[5] Sued O, Miro JM, Alquezar A, Claramonte X, Garcia F, Plana M, Arnedo M, de Lazzari E, Gil C, Manzardo C, Blanco JL, Martínez E, Mallolas J, Joseph J, Pumarola T, Gallart T, Gatell JM (2006). Primary human immunodeficiency virus type 1 infection: clinical, virological and immunological characteristics of 75 patients (1997–2003). Enferm Infecc Microbiol Clin.

[6] Pena JM, Martinez-Lopez MA, Arnalich F, Barbado FJ, Vazquez JJ (1991). Esophageal candidiasis associated with acute infection due to human immunodeficiency virus: case report and review. Rev Infect Dis.

[7] Hong KW, Kim SI, Kim YJ, Wie SH, Kim YR, Yoo JH, Han NI, Kang MW (2011). Acute cytomegalovirus pneumonia and hepatitis presenting during acute HIV retroviral syndrome. Infection.

[8] Moss PJ, Read RC, Kudesia G, McKendrick MW (1995). Prolonged cryptosporidiosis during primary HIV infection. J Infect.

[9] Silva Mde O, Bastos M, Netto EM, Gouvea NA, Torres AJ, Kallas E, Watkins DI, Altfeld M, Brites C (2010). Acute HIV infection with rapid progression to AIDS. Braz J Infect Dis.

[10] Signorini L, Gulletta M, Coppini D, Donzelli C, Stellini R, Manca N, Carosi G, Matteelli A (2007). Fatal disseminated toxoplasmosis during primary HIV infection. Curr HIV Res.

[11] Vento S, Di Perri G, Garofano T, Concia E, Bassetti D (1993). Pneumocystis carinii pneumonia during primary HIV-1 infection. Lancet.

[12] Joh JS, Lee CH, Lee JE, Park YK, Bai GH, Kim EC, Han SK, Shim YS, Yim JJ (2007). The interval between initiation of anti-tuberculosis treatment in patients with culture-positive pulmonary tuberculosis and receipt of drug-susceptibility test results. J Korean Med Sci.

[13] Huyen MN, Tiemersma EW, Lan NT, Cobelens FG, Dung NH, Sy DN, Buu TN, Kremer K, Hang PT, Caws M, O'Brien R, van Soolingen D (2010). Validation of the GenoType MTBDRplus assay for diagnosis of multidrug resistant tuberculosis in South Vietnam. BMC Infect Dis.

[14] Jenkins HE, Crudu V, Soltan V, Ciobanu A, Domente L, Cohen T (2014). High risk and rapid appearance of multidrug resistance during tuberculosis treatment in Moldova. Eur Respir J.

[15] Merker M, Kohl TA, Roetzer A, Truebe L, Richter E, Rusch-Gerdes S, Fattorini L, Oggioni MR, Cox H, Varaine F, Niemann S (2013). Whole genome sequencing reveals complex evolution patterns of multidrug-resistant Mycobacterium tuberculosis Beijing strains in patients. PLoS One.

[16] Saunders NJ, Trivedi UH, Thomson ML, Doig C, Laurenson IF, Blaxter ML (2011). Deep resequencing of serial sputum isolates of Mycobacterium tuberculosis during therapeutic failure due to poor compliance reveals stepwise mutation of key resistance genes on an otherwise stable genetic background. J Infect.

[17] Pawlowski A, Jansson M, Skold M, Rottenberg ME, Kallenius G (2012). Tuberculosis and HIV co-infection. PLoS Pathog.

[18] Gandhi NR, Andrews JR, Brust JC, Montreuil R, Weissman D, Heo M, Moll AP, Friedland GH, Shah NS (2012). Risk factors for mortality among MDR- and XDR-TB patients in a high HIV prevalence setting. Int J Tuberc Lung Dis.

[19] Mesfin YM, Hailemariam D, Biadglign S, Kibret KT (2014). Association between HIV/AIDS and multi-drug resistance tuberculosis: a systematic review and meta-analysis. PLoS One.

[20] Naing C, Mak JW, Maung M, Wong SF, Kassim AI (2013). Meta-analysis: the association between HIV infection and extrapulmonary tuberculosis. Lung.

[21] Geldmacher C, Zumla A, Hoelscher M (2012). Interaction between HIV and Mycobacterium tuberculosis: HIV-1-induced CD4 T-cell depletion and the development of active tuberculosis. Curr Opin HIV AIDS.

[22] Byers DK, Decker CF (2008). Unusual case of Pneumocystis jiroveci pneumonia during primary HIV infection. AIDS Read.

[23] Lin PL, Flynn JL (2010). Understanding latent tuberculosis: a moving target. J Immunol.

[24] Wells CD, Cegielski JP, Nelson LJ, Laserson KF, Holtz TH, Finlay A, Castro KG, Weyer K (2007). HIV infection and multidrug-resistant tuberculosis: the perfect storm. J Infect Dis.

[CR25] The pre-publication history for this paper can be accessed here:http://www.biomedcentral.com/1471-2334/14/462/prepub

