# Supplementary figures and images for: Dissemination of multidrug-resistant tuberculosis in a patient with acute HIV infection
Source: BMC Infect Dis. 2014 Aug 26;14:462. doi: 10.1186/1471-2334-14-462 (PMC4156626; doi:10.1186/1471-2334-14-462)

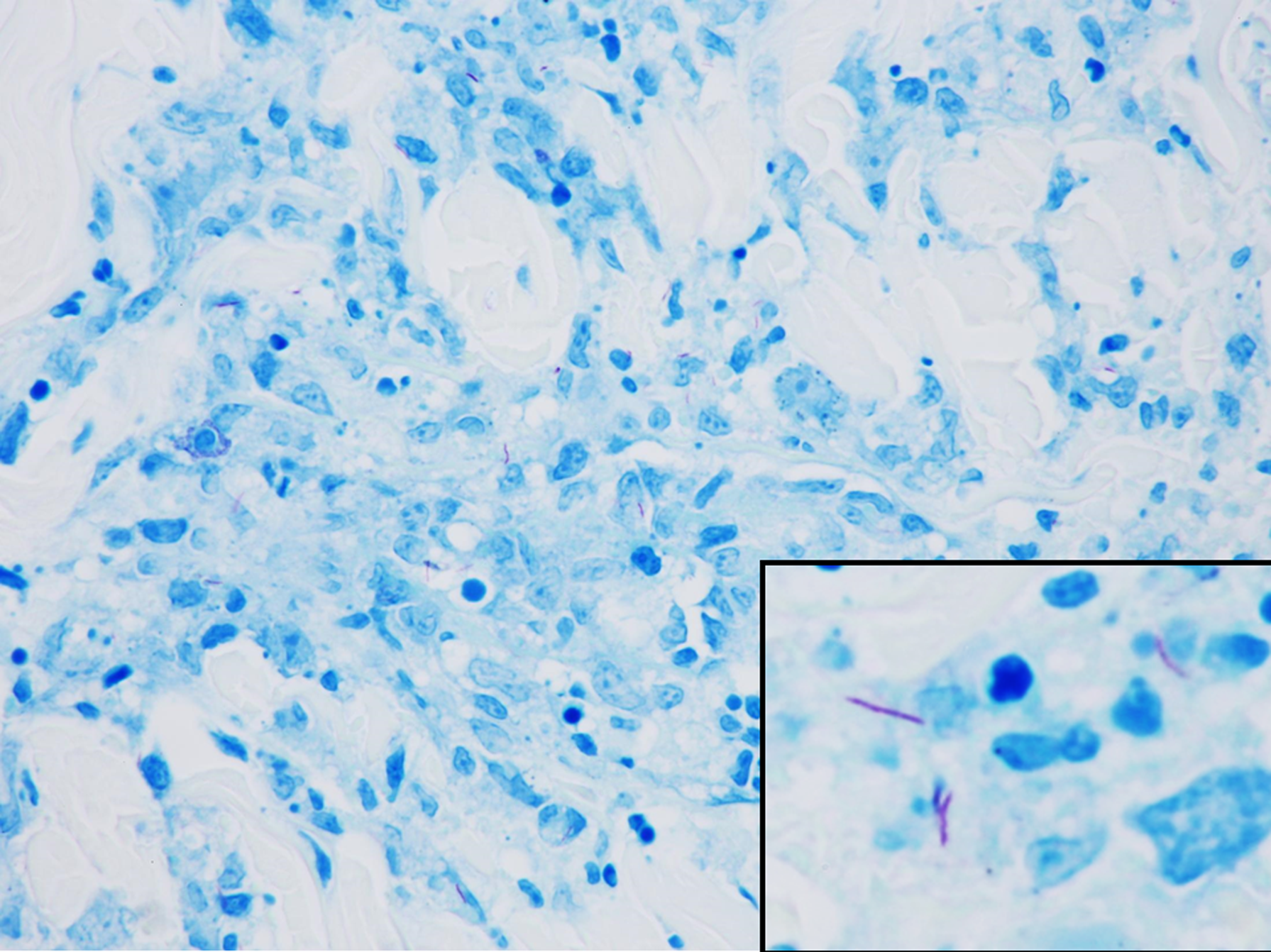

Supplement: Supplementary file 1 — Authors’ original file for figure 1 [file 12879_2014_3765_MOESM1_ESM.tif]
